# Curcumin Ameliorates the Reduction Effect of PGE_2_ on Fibrillar β-Amyloid Peptide (1-42)-Induced Microglial Phagocytosis through the Inhibition of EP2-PKA Signaling in N9 Microglial Cells

**DOI:** 10.1371/journal.pone.0147721

**Published:** 2016-01-29

**Authors:** Gen-Lin He, Zhen Luo, Ju Yang, Ting-ting Shen, Yi Chen, Xue-Sen Yang

**Affiliations:** Department of Tropic Hygiene, Institute of Tropical Medicine, Third Military Medical University, Chongqing, People’s Republic of China; University of Cologne, GERMANY

## Abstract

Inflammatory activation of microglia and β amyloid (Aβ) deposition are considered to work both independently and synergistically to contribute to the increased risk of Alzheimer’s disease (AD). Recent studies indicate that long-term use of phenolic compounds provides protection against AD, primarily due to their anti-inflammatory actions. We previously suggested that phenolic compound curcumin ameliorated phagocytosis possibly through its anti-inflammatory effects rather than direct regulation of phagocytic function in electromagnetic field-exposed N9 microglial cells (N9 cells). Here, we explored the prostaglandin-E_2_ (PGE_2_)-related signaling pathway that involved in curcumin-mediated phagocytosis in fibrillar β-amyloid peptide (1–42) (fAβ_42_)-stimulated N9 cells. Treatment with fAβ_42_ increased phagocytosis of fluorescent-labeled latex beads in N9 cells. This increase was attenuated in a dose-dependent manner by endogenous and exogenous PGE_2_, as well as a selective EP2 or protein kinase A (PKA) agonist, but not by an EP4 agonist. We also found that an antagonist of EP2, but not EP4, abolished the reduction effect of PGE_2_ on fAβ_42_-induced microglial phagocytosis. Additionally, the increased expression of endogenous PGE_2_, EP2, and cyclic adenosine monophosphate (AMP), and activation of vasodilator-stimulated phosphoprotein, cyclic AMP responsive element-binding protein, and PKA were depressed by curcumin administration. This reduction led to the amelioration of the phagocytic abilities of PGE_2_-stimulated N9 cells. Taken together, these data suggested that curcumin restored the attenuating effect of PGE_2_ on fAβ_42_-induced microglial phagocytosis via a signaling mechanism involving EP2 and PKA. Moreover, due to its immune modulatory effects, curcumin may be a promising pharmacological candidate for neurodegenerative diseases.

## Introduction

Alzheimer’s disease (AD) is the foremost form of dementia, and is increasing as the population ages. It is defined by two cardinal pathologic features: senile plaques and neurofibrillary degeneration [1]. Inexplicably, most cases of AD are associated with decreased clearance and degradation of amyloid beta (Aβ) [2] and increased secretion of inflammatory mediators, both associated with the phenotypic activation of microglial cells. It is widely accepted that beneficial strategies against AD may be attributabled to the promotion of phagocytosis and inhibition of the pro-inflammatory response in microglia.

Chronic inflammation has long been hypothesized to be a driving force in promoting the development of AD, leadings to increasing studies for exploring whether inflammatory products have a direct or indirect effect on Aβ clearance. In addition, it is important to investigate the pro-inflammatory mediators that regulate the clearance of Aβ by microglia. Prior studies have shown that pro-inflammatory cytokines act selectively to regulate microglial phagocytosis and Aβ load [3, 4]. Additional evidences have revealed that inducible isoform cyclooxygenase 2 (COX-2)-derived prostaglandin (PG) E_2_ mediates potentiation of the inflammatory response and amyloid plaque formation [5, 6]. Moreover, it has been proposed that microglial PGE_2_ receptor subtype 2 (EP2) signaling contributes to Aβ plaque burden in AD transgenic mice [7, 8], and that EP2 signaling suppresses microglial phagocytosis of Aβ_42_ in primary microglia cultures [9, 10]. These studies suggest that pharmacologic compounds targeting microglial EP2 would be an effective therapeutic option for AD.

Several studies demonstrate that natural compounds limiting neuroinflammation and promoting Aβ clearance may be more efficacious at ameliorating microglia-associated neurodegenerative diseases. Among these immuno-modulators, curcumin is the active compound in turmeric (*Curcuma longa*), an herbal medicine that has been widely used for many centuries in India and China [11, 12]. Recent experimental data further support the therapeutic potential of curcumin in the pathophysiology of AD. Curcumin proved to be immunomodulatory, inhibiting neurotoxicity and increasing the index of phagocytosis [13, 14]. In a previous work, we demonstrated that curcumin may ameliorate defective microglial phagocytosis via its anti-inflammatory effects, and not by direct regulation of phagocytic function in electromagnetic field-exposed N9 cells [15]. Although several cytokines have been shown to modulate phagocytic ability of microglia *in vitro* [3, 16], the exact pro-inflammatory molecule underlying the salutary effect of curcumin on microglial phagocytosis in AD is unidentified.

Recent evidence suggests that curcumin inhibits the production of microglia-derived PGE_2_ in response to inflammatory stimulation [17]. Given the fact that PGE_2_ is highly released in the AD brain [5, 6] and has a depressed effect on microglial phagocytosis [7, 8], we hypothesized that curcumin regulates microglial phagocytosis via PGE_2_ and its related signaling pathway. Herein, we first tested whether both exogenous and endogenous PGE_2_ are involved in immunomodulatory phagocytosis in fAβ_42_-stimulated N9 microglial cells (N9 cells). We then evaluated the ability of curcumin to ameliorate phagocytic abilities of PGE_2_ and fAβ_42_-stimulated N9 cells. Our results demonstrated that curcumin positively regulates microglial phagocytotic activity through inhibition of PGE_2_-EP2 signaling in Aβ_42_-stimulated N9 cells. The results may provide critical information supporting the therapeutic use of curcumin in neurologic disorders associated with activated microglia.

## Materials and Methods

### Cell culture and treatment

The immortalized murine microglial cell line N9 was a gift from Dr. Yun Bai (Department of Genetics, Third Military Medical University, China), and was original established by immortalization of day 13 embryonic brain cultures with the 3RV retrovirus carrying an activated v-myc oncogene as previously described [18, 19]. Briefly, cells were grown in Iscove's modified Dulbecco's medium (IMDM; HyClone, Logan, UT, USA) supplemented with 10% heat-inactivated fetal bovine serum (FBS; HyClone), 2 mM glutamine, 100 U/ml penicillin, 100 μg/ml streptomycin, and 50 μM 2-mercaptoethanol (Sigma-Aldrich, St. Louis, MO, USA). Cells were seeded in 25 cm^2^ T-flasks (5×10^6^ cells/flask), 6-well plates (5×10^5^ cells/well), 24-well plates (1.5×10^5^ cells/well) or 96-well plates (1×10^4^ cells/well) at 37°C in a humidified 5% CO_2_ atmosphere. N9 cells were passaged every three days with 1:4 split ratio and used at passages 3–10.

After 24 h incubation, cell culture medium was replaced with serum-free IMDM supplemented with the compounds of interest, and incubated for 15 or 30 min at 37°C. Synthetic β-amyloid peptide (1–42) (Aβ_42_; GL Biochem, Shanghai, China) was incubated at 37°C for 7 days in medium to promote fibril formation. Pharmacologic agents used in different experiments included a solvent control (tissue culture grade dimethylsulfoxide (DMSO), 30 min; Sigma-Aldrich); curcumin (30 min, 5, 10 and 20 μM; Sigma-Aldrich); antagonists of PG receptors EP1-4 (GW848687X (5 μM), AH6809, L-798106 (Sigma-Aldrich), and GW627368X (each 15 min,10 μM; Cayman Chemicals, Ann Arbor, MI, USA, unless otherwise indicated)); inhibitor of PKA (H89 (30 min, 20 μM; Sigma-Aldrich)); agonists of EP1-4 (17-phenyl trinor Prostaglandin E2 (PTPE2), butaprost, sulprostone, and L-902,688 (5 μM) (each 15 min, 10 μM; Cayman Chemicals, unless otherwise indicated)); activator of PKA (Adenosine 3ʹ,5ʹ-cyclic Monophosphate, N^6^-Benzoyl-, Sodium Salt (6-Bnz-cAMP) (30 min, 500 μM; Calbiochem EMD Millipore Corporation, Billerica, MA, USA)). Doses of pharmacologic agents were chosen based on prior specificity studies, and were shown to not alter the growth characteristics of tested cell lines. Then, cells were treated with or without fibrillar Aβ peptide (1–42) (fAβ_42_, 1 and 5 μM; GL Biochem) and/or prostaglandin-E_2_ (PGE_2_, 1, 5 and 10 μM; Cayman Chemicals) for 1, 3, or 6 h before they were subjected to a 1 h process of phagocytosis of fluorescent-labeled latex beads, or to staining and protein extraction. To investigate the effect of endogenous expression of PGE_2_ on microglial phagocytosis, cells were treated with *Escherichia coli* LPS (0.1, 0.5 and 1 μg/ml; Sigma-Aldrich).

### Cell viability

Cell Counting Kit-8 assay (CCK-8; Dojindo, Shanghai, China) was used to indirectly assess cell viability dependent of several proliferation-related elements including dehydrogenase, NAD(H), NADP(H), and mitochondrial activity. Briefly, cells were seeded at a density of 3 × 10^3^ cells/well for 24 h or 5 × 10^3^ cells/well for 3 and 6 h in the well of 96-well plates in IMDM medium at 37°C and then cultured using various concentrations of fAβ_42_ and LPS. At the end of the culture period, 10 μl of CCK-8 solution was added to each well of the culture plate. After a 2-h incubation at 37°C, absorbance at 450 nm was measured with a plate reader (BioTek Epoch, Winooski, VT, USA). A control was performed in parallel to monitor the influence of IMDM medium on the assays. Cell viability was expressed as a percentage of the control cell culture value using the following formula: Cell viability = (absorption of sample—absorption of background)/ (absorption of control—absorption of background) × 100%.

### Phagocytosis assay

Phagocytosis was determined with latex beads (1 μm particular carboxylate-modified polystyrene, excitation/emission 470/540, Sigma-Aldrich), as previously described with slight modifications [10]. In brief, latex beads were diluted to 0.00125% with medium, and applied to N9 cells after sham or pharmacologic treatment in culture medium at 37°C for 1 h. After incubation, a washing step with cold serum-free medium was performed to interrupt any interaction between phagocytosing microglia and uningested beads. The phagocytic ability of N9 cells was evaluated using the fluorescence intensity of the engulfed beads on a flow cytometer and fluorescence microscopy. In the fluorescence-activated cell sorting (FACS) analysis, cells were collected and washed three times with ice-cold phosphate-buffered saline (PBS), and resuspended in 250 μl ice-cold PBS. The cell suspension was applied to an Accuri^™^ C6 flow cytometer (BD Biosciences, San Jose, CA). FACS analysis of 10,000 events (cells) was collected for each sample. In the microscopy assay, the number of ingested beads per cell and the number of phagocytosed cells were counted to determine the mean number of ingested beads per cell, in a blinded fashion with a minimum of 500 cells per slide. Five replicates were used for each experimental condition. The positive control was N9 cells treated with beads alone. Images of phagocytosing cells were captured using a LSM 780 confocal laser scanning microscope (Carl Zeiss GmbH, Jena, Germany).

### Immunofluorescent staining

The cells were seeded on glass cover slips in the well of 24- well plates, and then washed three times with ice-cold PBS. Cells were then fixed with 4% paraformaldehyde for 30 min and permeabilized with 0.1% Triton X-100 in 1% BSA-containing PBS for 15 min at room temperature. After being fixed and permeabilized, the cells were blocked with goat serum (Zhongshan Goldenbridge Biotechnology (ZsBio), Beijing, China) for 20 min at room temperature and washed three times in PBS. To visualize the morphologic features of cells, cell cultures were incubated with rabbit anti-ionized calcium-binding adapter molecule 1 (Iba1, 1:200; Wako Pure Chemical Industries, Osaka, Japan) and a second antibody chicken anti-rabbit IgG-CF^™^ 633 (1:500; Sigma-Aldrich) for 1 h at 37°C in the dark. Cell cultures were then washed and mounted with aqueous-based anti-fade mounting medium. Fluorescence was detected with a LSM 780 confocal laser scanning microscope (Carl Zeiss GmbH, Jena, Germany).

### Enzyme immunoassay (EIA) of PGE_2_ and cAMP

Cells were seeded in 6-well plates. After pharmacologic treatment of N9 cells in culture medium, cell culture supernatants were collected and PGE_2_ levels quantified by EIA according to the manufacturer's instructions (Cayman Chemicals). For cAMP experiments, culture supernatants were aspirated and the cells were washed three times with ice-cold PBS, and re-suspended in 100 μl ice-cold PBS. 10 μl of the cell collections were quantified using a cell counter (TC20, Bio-Rad, Hercules, CA, USA). Immediately thereafter, 10 μl of 1N HCl was added to the remainder of the 90 μl collections, incubated for 10 min at room temperature, and centrifuged at 600 x g for 10 min at 2–8°C to remove cellular debris. The sample was neutralized with 10 μl of 1N NaOH. Subsequently, each sample was diluted two-fold with Calibrator Diluent RD5-55 and intracellular cAMP levels were determined immediately to minimize degradation, according to the manufacturer's instructions (R&D Systems, Minneapolis, MN, USA).

### Immunoblot analysis

Cells were washed with ice-cold PBS and scraped in RIPA lysis buffer containing protease and phosphatase inhibitors (Roche, Penzberg, Germany). Whole cell extracts (80 μg/lane) were separated by 10% SDS-polyacrylamide gel and then transferred onto PVDF membranes (Bio-Rad). The membranes were blocked in PBS with 5% non-fat milk for 1 h and then incubated with primary antibodies recognizing EP2 (1:200; Cayman Chemicals), EP4 (1:100; Cayman Chemicals), phospho-CREB (Ser133, 1:1000; Cell Signaling Technology, Danvers, MA, USA), and phospho-VASP (Ser157, 1:500; Cell Signaling Technology). Membranes were washed four times for 5 min each in tris-buffered saline Tween-20 (TBST) and then incubated with horseradish peroxidase (HRP)-conjugated secondary antibodies (ZsBio) for 1 h at room temperature. After the incubation, the membranes were reacted with enhanced chemiluminescence reagent (Bio-Rad) and the signal was detected using a ChemiDoc MP gel imaging system (Bio-Rad). Glyceraldehyde 3-phosphate dehydrogenase (GAPDH, 1:1000; Cell Signaling Technology) was used as internal controls. Protein bands were semi-quantified by densitometric analysis using ImageJ 1.49.

### Statistical Analysis

Statistical analysis was performed using SPSS software. Each experiment was repeated a minimum of three times and the data expressed as means ± SEM. The normality of data was verified by Kolmogorov-Smirnov before further analysis. Statistical differences between the groups were assessed by one or two-way analysis of variance (ANOVA) followed by Tukey’s test. Statistical significance was established at *P* < 0.05, unless otherwise indicated.

## Results

### Effect of LPS, PGE_2_, and fAβ_42_ on the viability of N9 cells

**Fig 1:**
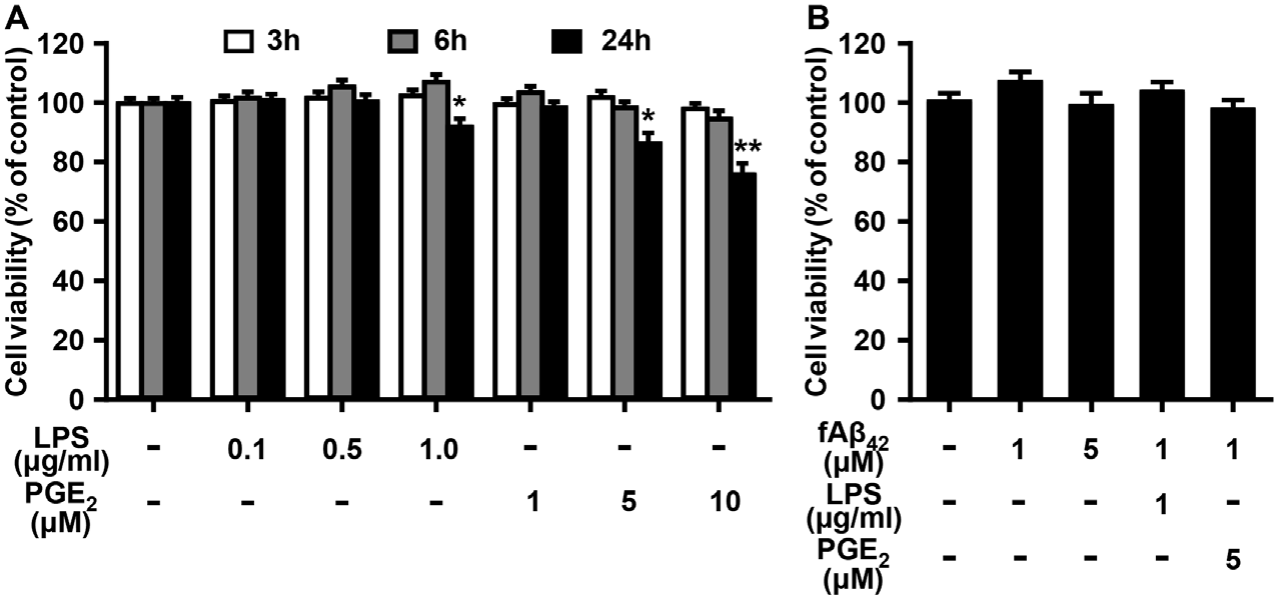
Effect of LPS, PGE_2_, and/or fAβ_42_ on cell viability in cultured N9 cells.

Cell viability of N9 cells was indirectly assessed in the dose-response and time-course stimulation of the LPS and PGE_2_ by proliferation-based CCK-8 assay. LPS and PGE_2_ were shown to be cytotoxic to N9 cells with a concentration of 1 μg/ml and 5 μM, respectively (Fig 1A). No significant changes in cell viability were determined at the 3 and 6 h points of LPS and PGE_2_ treatment. There were significant changes in cell survival when N9 cells were exposed to 1 μg/ml LPS and 5 or 10 μM PGE_2_ for 24 h. We then measured the effect of fAβ_42_ on N9 cell viability. Not surprisingly, fAβ_42_ in the concentration of 1–5 μM did not affect the viability of N9 cells for 6 h treatment (Fig 1B), which is in line with previous reports demonstrating that fAβ_42_ at the concentration of 1–5 μM will maintain the viability and enhanced microglial phagocytic function of microglial cells *in vitro* [10, 20]. Moreover, combined treatment of fAβ_42_ with LPS or PGE_2_ for 6 h also had no effect on cell viability of N9 cells (Fig 1B). These findings suggest that cell viability was not affected within 6 h treatment of LPS, PGE_2_, and fAβ_42_ treatment at the given concentration in microglial cells.

N9 cells were stimulated with LPS (0.1, 0.5, and 1 μg/ml) and PGE_2_ (1, 5, and 10 μM) in the presence or absence of fAβ_42_ (1 and 5 μM) for 3, 6, and 24 h. At the end of the incubation period, the CCK-8 assay was performed. (A) Dose response and time course effect of LPS and PGE_2_ on N9 cell viability. Statistical significance was determined by two-way ANOVA followed by Tukey’s test. (B) Dosage response and its combined effect with LPS or PGE_2_ for 6 h on N9 cell viability. Statistical significance was determined by One-way ANOVA followed by Tukey’s test. For (A) and (B) in each experimental condition, 5–8 replicate wells in a 96-well plate were tested. The results are expressed as % of the untreated control, and are presented as means ± standard error of the mean (SEM) of three independent experiments. *P <0.05, **P <0.01 vs. the untreated control group. con, control; LPS, lipopolysaccharide; PGE_2_, prostaglandin E2; fAβ42, fibrillar Aβ peptide (1–42).

### The reduction effect of PGE_2_ on fAβ_42_-induced phagocytosis in N9 cells

**Fig 2:**
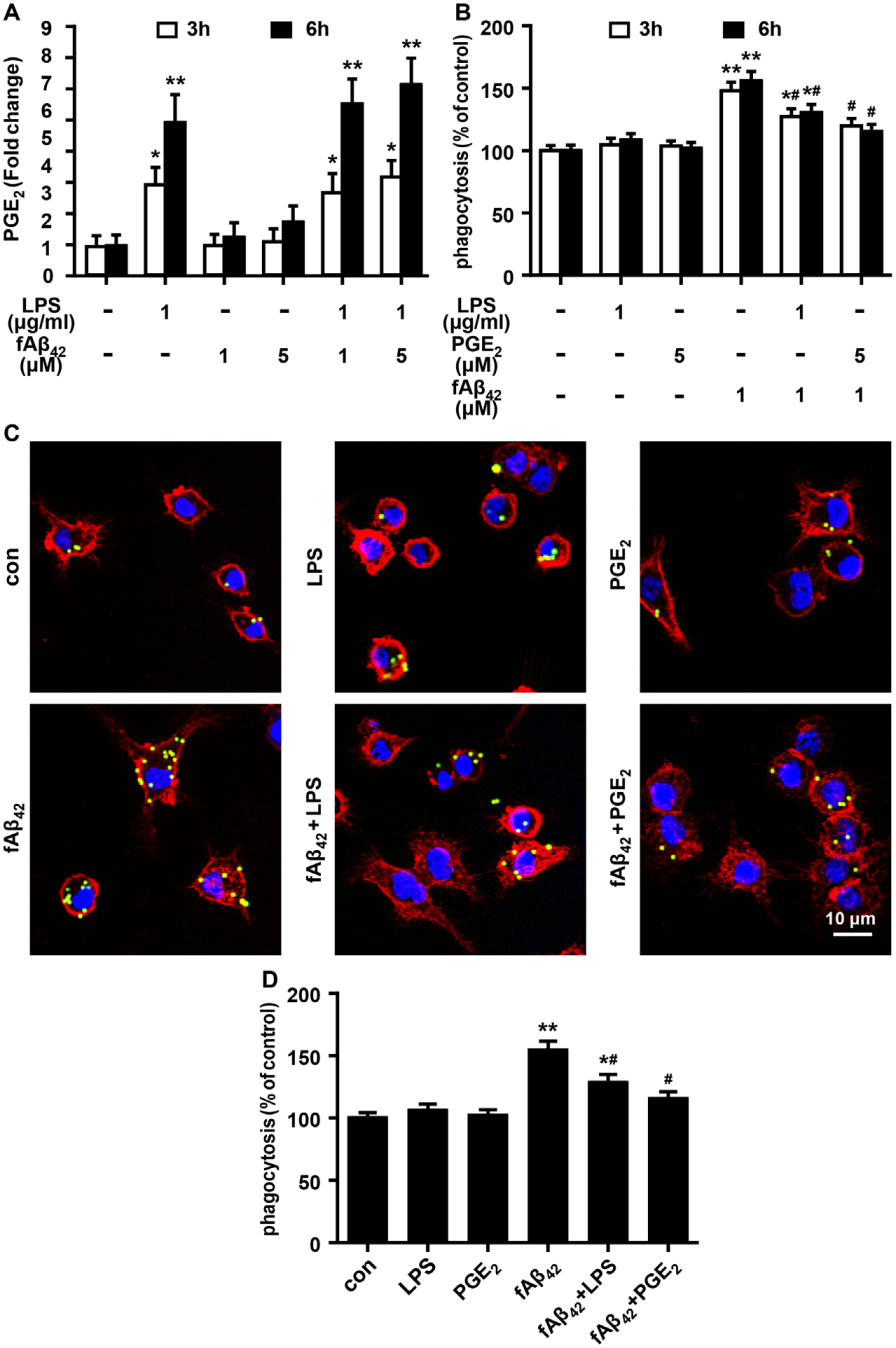
Effect of LPS and PGE_2_ on fAβ_42_-induced phagocytosis in N9 cells.

Given that Aβ_42_ fibrils are much less cytotoxic than oligomeric Aβ1–42 (oAβ_42_) in the induction of inflammation [20], we measured levels of PGE_2_ production in LPS and/or fAβ_42_-stimulated N9 cells, and found that fAβ_42_ treatment did not resulted in increase in PGE_2_ level, while LPS with or without fAβ_42_ treatment produced significantly higher levels of PGE_2_ (Fig 2A). These findings indicate the potential of LPS to mimic external stimuli to produce endogenous PGE_2_. To minimize the production of endogenous PGE_2_ induced by fAβ_42_, fAβ_42_ at a concentration of 1 μM was used in the following experiments of microglial phagocytosis, by using fluorescent-labeled latex beads to mimic the phagocytic model. As shown in Fig 2B, treatment with 1 μM fAβ_42_ for 3 and 6 h significantly increased phagocytosis of latex beads in N9 cells by FACS analysis. In addition, fAβ_42_-induced phagocytosis was dramatically reduced by exogenous PGE_2_ or LPS. Considering the rapid actions of PGE_2_ [21], LPS and PGE_2_ at the time point of 3 h served as the proper time point in the following experiment. Immunolocalization and confocal microscopy provided further evidence for a phagocytic response, showing a strong increase in phagocytic ability of fAβ_42_-stimulated N9 cells at 3 h (Fig 2C and 2D). Similarly, fAβ_42_-induced phagocytosis was reduced by exogenous PGE_2_ (5 μM) and LPS (1 μg/ml) at 3 h in N9 cells (Fig 2C and 2D). Moreover, cell cultures treated with PGE_2_, LPS or DMSO (S1 Fig) alone showed no significant changes in microglial phagocytosis in N9 cells cultured without fAβ_42_ treatment. Our observations confirm that PGE_2_ can act as a potential risk factor for impaired microglial phagocytosis.

N9 cells were stimulated with LPS (1 μg/ml) and PGE_2_ (5 μM) in the presence or absence of fAβ_42_ (1 and 5 μM) for 3 and 6 h. Then, cells were subjected to a 1 h process of phagocytosis of fluorescent-labeled latex beads (0.00125%) for the phagocytosis assay on a flow cytometer and fluorescence microscopy. (A) Enzyme immunoassay of PGE_2_ in N9 cells following the stimulation with LPS, fAβ_42_ (1 and 5 μM), individually and in combination. Experiments were performed with three replicates for each experimental condition. Data are presented relative to control and are presented as means ± SEM of five independent experiments. Statistical significance was determined by one-way ANOVA followed by Tukey’s test. (B) Average fluorescence intensity of latex beads ingested per group estimated using a flow cytometer. FACS analysis for each group were normalized compared as % of the untreated control. (C) Microscopy images of beads phagocytosis in N9 cells supplied with LPS and PGE_2_ in the presence or absence of fAβ_42_ (1 μM) for 3 h. Scale bar: 10 μm. (D) Quantification of phagocytosis. Phagocytic ratios were calculated from (C) as indicated in Methods. The results are normalized to the mean number of beads each cell engulfed (1 and 3 mean beads/cell were normalized as 100% and 150%, respectively). For (B) and (D), data are presented as means ± SEM of three independent experiments. Statistical significance was determined by two-way ANOVA followed by Tukey’s test. For (A), (B), and (D),*P < 0.05, **P <0.01 vs the untreated control group; ^#^*P* < 0.05 vs the fAβ_42_-stimulated group. con, control; LPS, lipopolysaccharide; PGE_2_, prostaglandin E2; fAβ_42_, fibrillar Aβ peptide (1–42).

### EP2 and EP4 Receptors is associated with phagocytic depression

**Fig 3:**
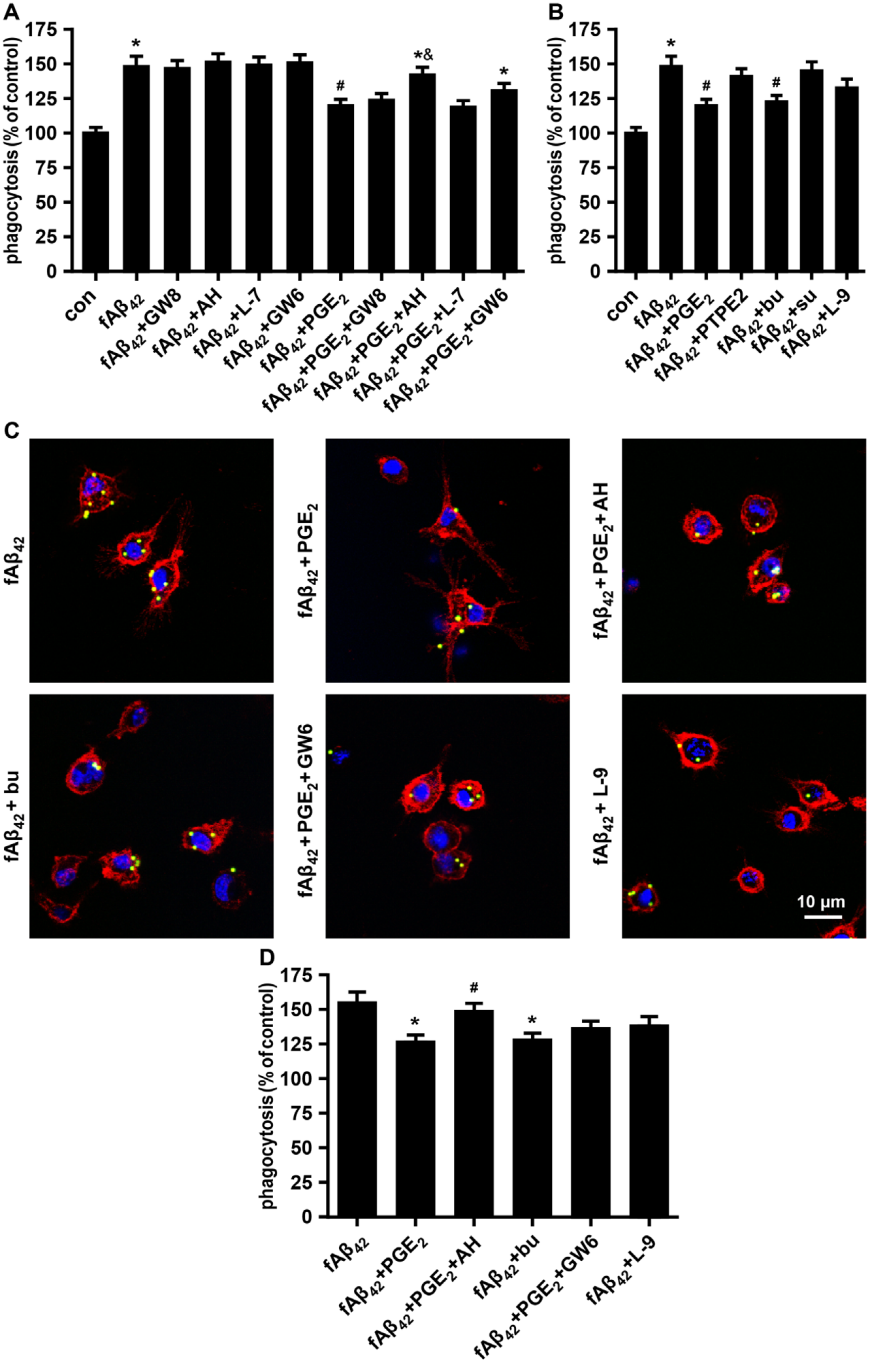
Effect of E-prostanoid receptors on fAβ_42_-induced phagocytosis with or without PGE_2_ in N9 cells.

PGE_2_-related changes in functional microglial activation may be blocked by inhibiting EP receptors. Accordingly, we performed a phagocytosis assay after blocking these receptors with selective antagonists. The FACS analysis indicated that the reduction effect of 5 μM PGE_2_ on fAβ42-induced phagocytosis was dramatically prevented by 10 μM AH6809 (EP2 antagonist), and was slightly prevented by 10 μM GW627368X (EP4 antagonist) (Fig 3A). In contrast, 5 μM GW848687X (EP1 antagonist) and 10 μM L-798106 (EP3 antagonist) showed no reversal effect of the reduced phagocytosis by the action of PGE_2_ in fAβ_42_-stimulated N9 cells. To further confirm the subtypes of the E-prostanoid receptor involved, cells were pretreated with EP1-4 agonists. It was found that 10 μM butaprost (EP2 agonist) exposure decreased the capacity of fAβ_42_-stimulated N9 cells to ingest latex beads, while 5 μM L-902,688 (EP4 agonist) was effective but not quite as potent (Fig 3B). No alteration of phagocytosis in N9 cells was observed with pretreatment of 10 μM 17-phenyl trinor prostaglandin E2 (PTPE2, EP1 agonist) and sulprostone (EP3 agonist). Preliminary tests were performed at different concentrations of these agents to establish the optimal dose (S2 and S3 Figs). We also found a similar pattern of phagocytosis function in N9 cells with pretreatment of the antagonist and agonist for EP2 and EP4, respectively, in the microscopy assay. PGE_2_-induced impaired microglial phagocytosis in fAβ_42_-stimulated N9 cells was prevented by AH6809, but not by GW627368X (Fig 3B, 3C and 3D). Fig 3C and 3D also shows a more robust decrease phagocytosis of beads in N9 cells with the pretreatment of 10 μM butaprost than 5 μM L-902,688. Taken together, these results demonstrated that the reduction effect of PGE_2_ on fAβ_42_-induced phagocytosis appeared to be mainly mediated by the EP2 receptor rather than the EP4 receptor.

N9 cells were pretreated with antagonists (A) or agonists (B) of PG receptors EP1-4. Then, cells were stimulated with fAβ_42_ (1 μM) in the presence or absence of exogenous PGE_2_ (5 μM) for 3 h. Subsequently, cells were subjected to a 1 h process of phagocytosis of fluorescent-labeled latex beads (0.00125%). Average fluorescence intensity of latex beads ingested and normalized phagocytosis analysis were estimated for each group using FACS analysis. (A) N9 cells were pretreated with antagonists of PG receptors EP1-4 (GW848687X (5 μM), AH6809 (10 μM), L-798106 (10 μM), and GW627368X (10 μM) (each 15 min)). (B) N9 cells were pretreated with agonists of EP1-4 (10 μM PTPE2, 10 μM butaprost, 10 μM sulprostone, and 5 μM L-902,688; 15 min each). (C) Microscopy images of beads phagocytosis in N9 cells supplied with antagonists or agonists of EP2 and EP4 for 15 min prior to fAβ_42_ (1 μM) in the presence or absence of PGE_2_ (5 μM). Scale bar: 10 μm. (D) Quantification of phagocytosis. Phagocytic ratios were calculated from (C) as indicated in Methods. For (A) and (B), the results are expressed as % of the untreated control. For (D), the results are normalized to the mean number of beads each cell engulfed (1 and 3 mean beads/cell were normalized as 100% and 150%, respectively). Data are presented as means ± SEM of three independent experiments. Statistical significance was determined by one-way ANOVA followed by Tukey’s test. For (A) and (B), *P < 0.05 vs the untreated control group; ^#^*P* < 0.05 vs the fAβ_42_-stimulated group; ^&^*P* < 0.05 vs the fAβ_42_ plused PGE_2_-stimulated group. For (D), *P < 0.05 vs the fAβ_42_-stimulated group; ^#^*P* < 0.05 vs the fAβ_42_ plused PGE_2_-stimulated group. con, control; PGE_2_, prostaglandin E2; fAβ_42_, fibrillar Aβ peptide (1–42); GW8, GW848687X; AH, AH6809; L-7, L-798106; GW6, GW627368X; PTPE2, 17-phenyl trinor Prostaglandin E2; bu, butaprost; su, sulprostone; L-9, L-902,688.

### Curcumin ameliorates EP2-dependent phagocytic depression

**Fig 4:**
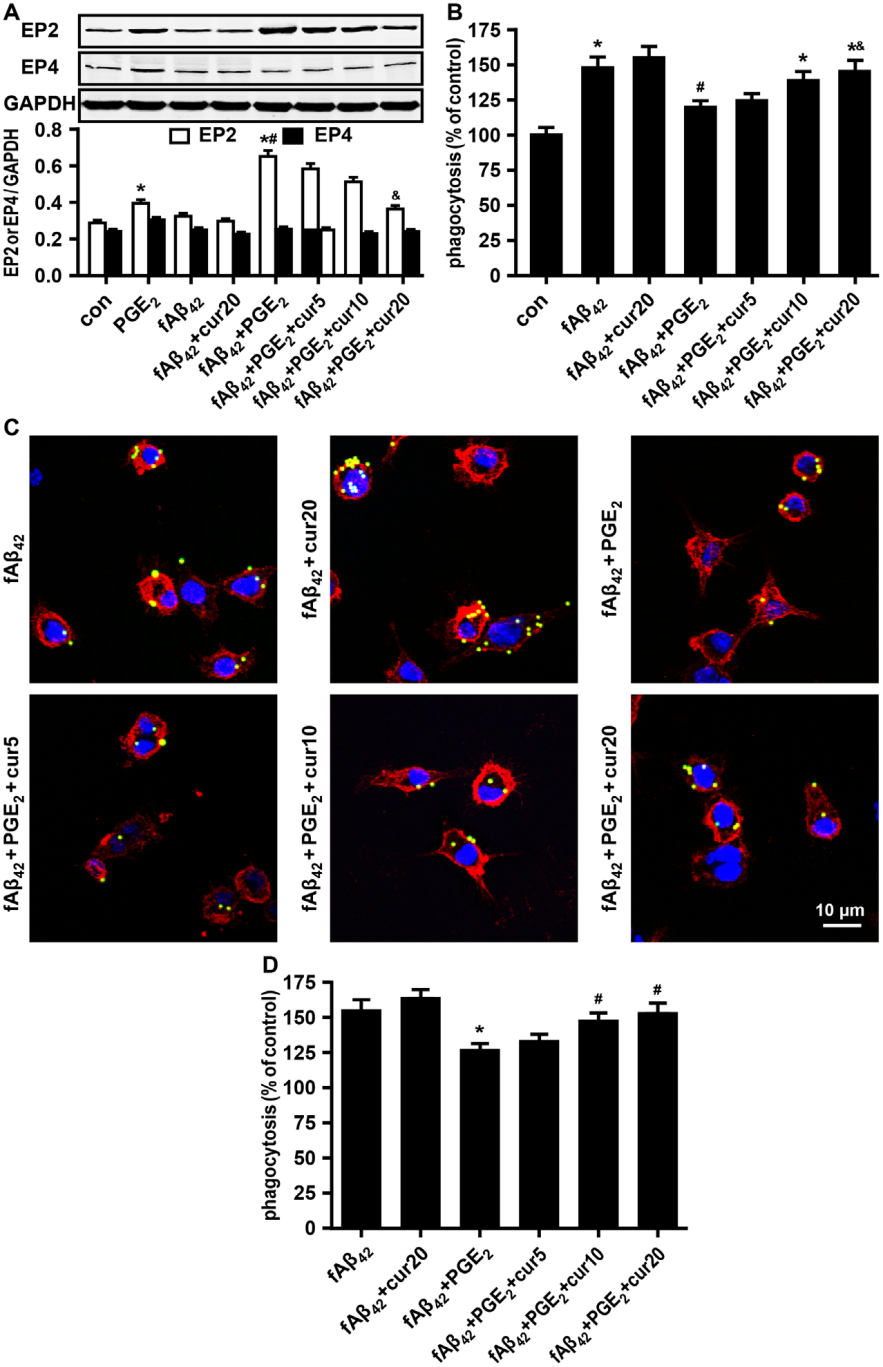
Effect of curcumin on fAβ_42_-induced phagocytosis with or without PGE_2_ in N9 cells.

The data above demonstrate that PGE_2_ inhibits phagocytosis in partial enhanced EP receptors in fAβ_42_-stimulated N9 cells, while EP2-dependent signaling activation is sufficient to suppress phagocytosis. We found that PGE_2_ significant increase the expression of EP2. Corresponding to no change of levels of PGE_2_ production in fAβ_42_ and/or curcumin-treated N9 cells (Fig 2A and S4 Fig), expression of EP2 and EP4 was not changed in fAβ_42_-stimulated N9 cells at 3 h (Fig 4A). A decreasing trend of fold change was found for EP2 expression by curcumin preconditioning in PGE_2_ and fAβ_42_ co-treated N9 cells (Fig 4A). Immunoblotting showed no change in EP4 expression in each group. We also investigated the effect of curcumin on improving microglial phagocytic function. Clearly, curcumin pretreatment concomitantly improves microglial phagocytosis in a dose-dependent manner against the reduction effect of PGE_2_ on fAβ_42_-induced phagocytosis in N9 cells (Fig 4B). Confocal microscopy provided further evidence for the amelioration of phagocytosis, showing a dose-dependent restoration of phagocytic ability by curcumin in PGE_2_ and fAβ_42_ co-treated N9 cells (Fig 4C and 4D). However, curcumin alone did not enhance microglial phagocytosis induced by fAβ_42_ (Fig 4B, 4C and 4D), or decrease EP2 expression in the control group (Fig 4A). These results suggest that EP2 signaling might be involved in the curcumin-mediated improvement of inhibition induced by PGE_2_ on fAβ_42_-stimulated microglial phagocytosis.

N9 cells were pretreated with curcumin (5, 10, and 20 μM) for 30 min prior to fAβ_42_ (1 μM) treatment in the presence or absence of exogenous PGE_2_ (5 μM) for 3 h. Then, cells were subjected to a 1 h process of phagocytosis of fluorescent-labeled latex beads (0.00125%). (A) Western blot quantification of EP2 and EP4. Curcumin significantly depressed the expression of EP2 in a dose-dependent manner 3 h after treatment with fAβ_42_ (1 μM) in the presence or absence of PGE_2_ (5 μM). (B) Curcumin concomitantly improves microglial phagocytosis in a dose-dependent manner 3 h after fAβ_42_ (1 μM) treatment in the presence or absence of PGE_2_ (5 μM). Average fluorescence intensity of latex beads ingested and normalized phagocytosis analysis were estimated for each group using FACS analysis. (C) Beads phagocytosis of N9 cells was restored by curcumin pre-conditioning for the phagocytosis assay by fluorescence microscopy. Scale bar: 10 μm. (D) Quantification of phagocytosis. Phagocytic ratios were calculated from (C) as indicated in Methods. For (B), the results are expressed as % of the untreated control. For (D), the results are normalized to the mean number of beads each cell engulfed (1 and 3 mean beads/cell were normalized as 100% and 150%, respectively). All data are presented as means ± SEM of three independent experiments. Statistical significance was determined by one-way ANOVA followed by Tukey’s test. For (A) and (B), *P < 0.05 vs the untreated control group; ^#^*P* < 0.05 vs the fAβ_42_-stimulated group. ^&^*P* < 0.05 vs the fAβ_42_ plused PGE_2_-stimulated group. For (D), *P < 0.05 vs the fAβ_42_-stimulated group; ^#^*P* < 0.05 vs the fAβ_42_ plused PGE_2_-stimulated group. con, control; PGE_2_, prostaglandin E2; fAβ_42_, fibrillar Aβ peptide (1–42); cur, curcumin.

### Curcumin ameliorates phagocytosis by inhibition of cAMP increase and PKA activation

**Fig 5:**
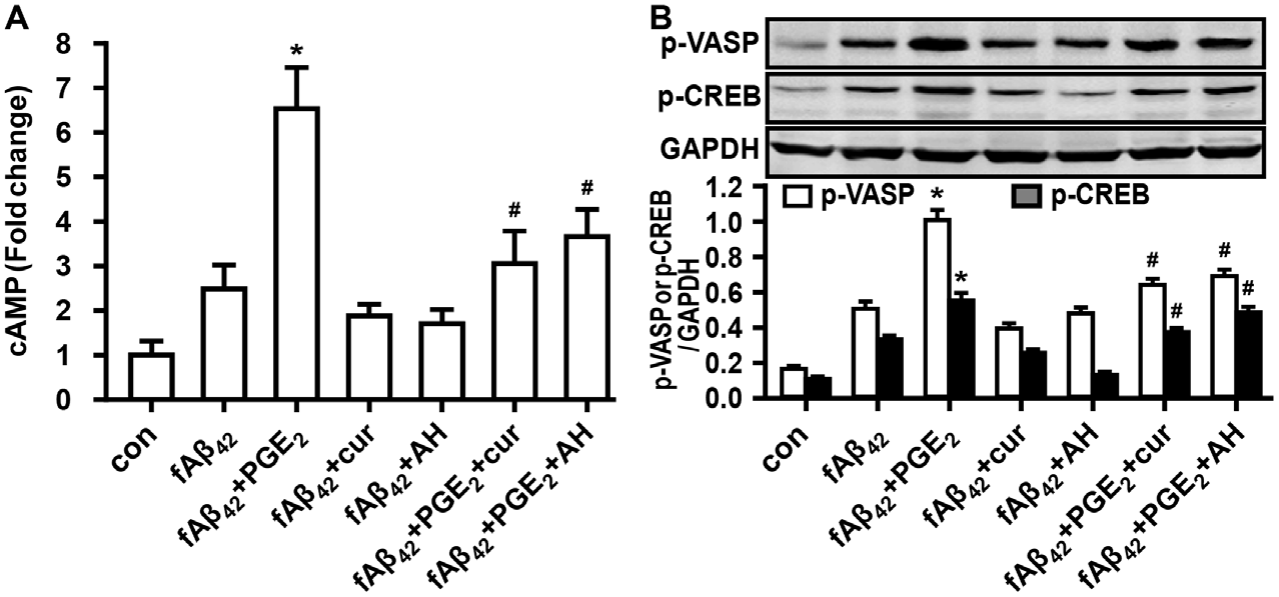
Involvement of cAMP-PKA activity in the restoration of PGE_2_ impaired phagocytiosis of N9 cells incubated in curcumin.

It is well known that both EP2 and EP4 receptors stimulate an increase in cAMP in various immune cells. Our results demonstrated that PGE_2_ caused a significant enhancement of increased levels of intracellular cAMP, which was blocked by pretreatment of 10 μM AH 6809 and 20 μM curcumin in fAβ_42_-stimulated N9 cells (Fig 5A). Meanwhile, cells pretreated with AH 6809 or curcumin alone showed a low level of cAMP in fAβ_42_-stimulated cell groups (Fig 5A), showing similar none affected pattern of fAβ_42_-induced phagocytosis with the pretreatment of AH 6809 or curcumin alone in the aforementioned description (Figs 3A, 4B and 4C). Given the above results, we focused our studies on the binding protein of cAMP, *i*.*e*., the PKA effector. Because the phosphorylation of vasodilator-stimulated phosphoprotein (VASP) is a typical surrogate of PKA activity [22, 23], we assessed the phosphorylation of VASP in the restoration of PGE_2_ impaired phagocytiosis of N9 cells incubated in curcumin. We also investigated the downstream phosphorylation of the transcription factor CREB for PKA. The immunoblot analysis revealed that the VASP phosphorylation and downstream CREB signaling were increased in fAβ_42_-stimulated N9 cells with PGE_2_ preconditioning. The addition of curcumin modified the expression of p-VASP and p-CREB in the inhibitory effect induced by PGE_2_ on fAβ_42_-stimulated N9 cells (Fig 5B).

N9 cells were pretreated with curcumin (20 μM) for 30 min or AH6809 (10 μM) for 15 min prior to fAβ_42_ (1 μM) treatment in the presence or absence of exogenous PGE_2_ (5 μM) for 3 h. (A) Cells were lysed and increase of intracellular cAMP was measured by enzyme immunoassay. Experiments were performed with three replicates for each experimental condition. Data are presented relative to control and are presented as means ± SEM of five independent experiments. (B) Cells were lysed and immunoblot analysis was performed for p-VASP, a marker of PKA activation, and p-CREB, which is a selectively activated downstream signaling factor of PKA. Data are presented as means ± SEM of three independent experiments. For (A) and (B), statistical significance was determined by one-way ANOVA followed by Tukey’s test. *P < 0.05 vs the fAβ_42_-stimulated group; ^#^*P* < 0.05 vs the fAβ_42_ plused PGE_2_-stimulated group. con, control; PGE_2_, prostaglandin E2; fAβ_42_, fibrillar Aβ peptide (1–42); cur, curcumin; cAMP, cyclic adenosine monophosphate; PKA, protein kinase A; p-VASP, vasodilator-stimulated phosphoprotein; CREB, cyclic AMP responsive element-binding protein.

**Fig 6:**
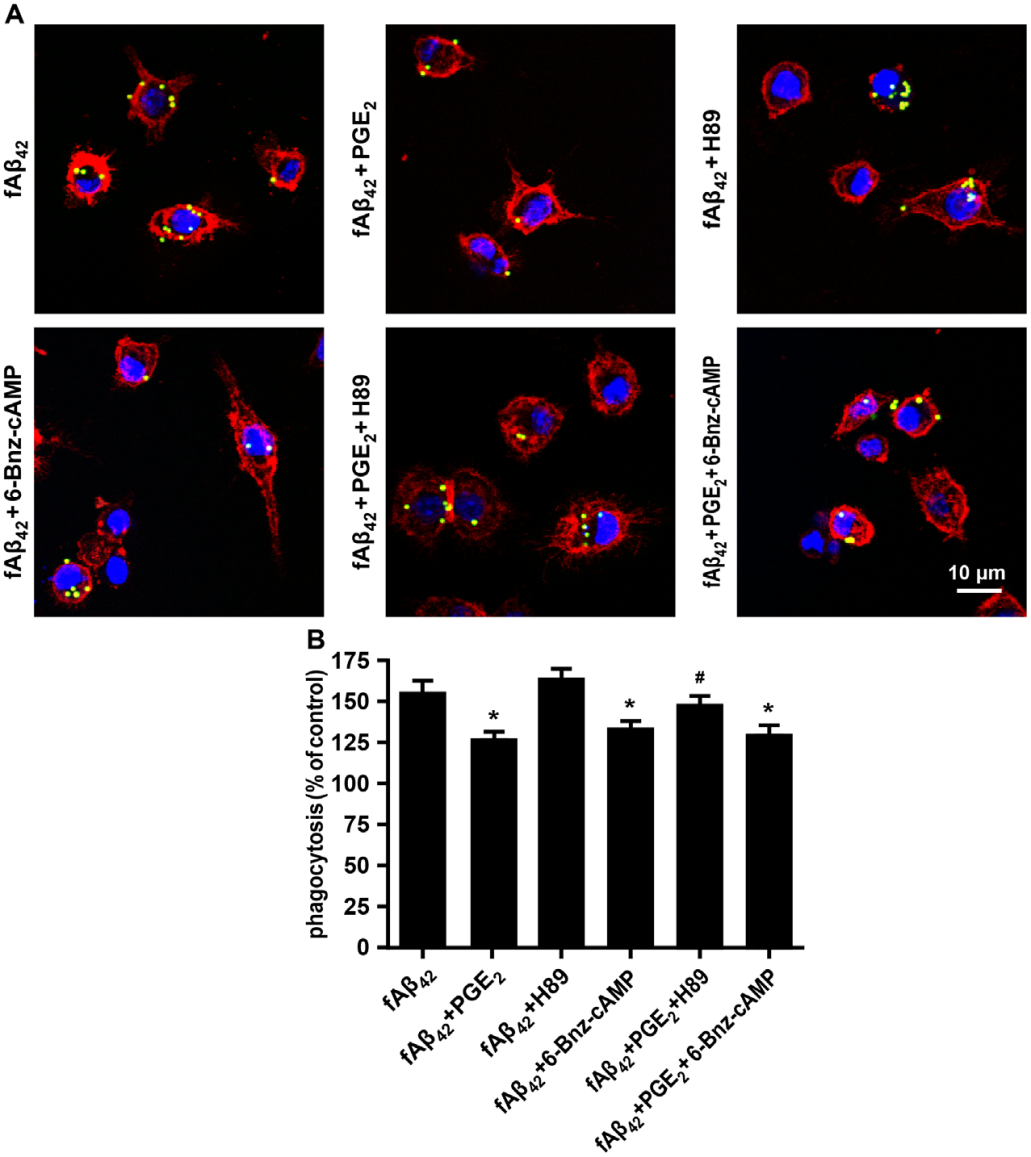
PGE_2_-mediated inhibition of phagocytos in fAβ_42_-stimulated N9 cells is PKA dependent.

To further test whether PKA activation mediated the actions of cAMP on the inhibitory effect of PGE_2_ in fAβ_42_-stimulated N9 cells, various concentrations of H89 (PKA inhibitor) and adenosine 3ʹ,5ʹ-cyclic monophosphate, N^6^-Benzoyl-, sodium salt (6-Bnz-cAMP) (PKA activator) were used. Pharmacologic studies showed that 20 μM H89 abolished the inhibitory effect of PGE_2_ on fAβ_42_-stimulated microglial phagocytosis, while 500 μM 6-Bnz-cAMP significant reduced fAβ_42_-induced phagocytosis of latex beads in N9 cells. (Fig 6A, 6B and S5 Fig), suggesting the partial involvement of PKA activity in PGE_2_-induced impaired phagocytosis via EP2. In contrast, H89 alone did not affect microglial phagocytosis induced by fAβ_42_. Taken together, these results indicate that curcumin may restore the inhibitory effect of PGE_2_ on Aβ_42_-induced microglial phagocytosis via a signaling mechanism involving EP2 and PKA.

N9 cells were pretreated with PKA inhibitor H89 (20 μM) or PKA activator 6-Bnz-cAMP (500 μM) for 30 min. Then, cells were stimulated with fAβ_42_ (1 μM) in the presence or absence of exogenous PGE_2_ (5 μM) for 3 h. Subsequently, cells were subjected to a 1 h process of phagocytosis of fluorescent-labeled latex beads (0.00125%). (A) Microscopy images of bead phagocytosis in N9 cells were captured using a confocal laser scanning microscope. Scale bar: 10 μm. (B) Quantification of phagocytosis. Phagocytic ratios were calculated from (A) as indicated in Methods. The results are normalized to the mean number of beads each cell engulfed (1 and 3 mean beads/cell were normalized as 100% and 150%, respectively). Data are presented as means ± SEM of three independent experiments. Statistical significance was determined by two-way ANOVA followed by Tukey’s test. *P < 0.05 vs the fAβ_42_-stimulated group; ^#^*P* < 0.05 vs the fAβ_42_ plused PGE_2_-stimulated group. con, control; PGE_2_, prostaglandin E2; fAβ_42_, fibrillar Aβ peptide (1–42); cur, curcumin; PKA, protein kinase A; 6-Bnz-cAMP, Adenosine 3ʹ,5ʹ-cyclic Monophosphate, N^6^-Benzoyl-, Sodium Salt.

## Discussion

In the present study, we observed the immunoregulation of microglial phagocytic ability by the inflammatory mediator PGE_2_ in fAβ_42_-stimulated N9 cells. We found a significant decrease of fAβ_42_-activated microglial phagocytosis of fluorescent-labeled latex beads by exogenous PGE_2_ or LPS-induced endogenous PGE_2_. Our data is in agreement with the classic story that impaired phagocytosis and increased expression of PGE_2_ occurs in the brains of AD patients, suggesting a failure in the phagocytic clearance ability in a PGE_2_-related chronic inflammatory environment. Hence, the pro-inflammatory PGE_2_ appears to have a nonredundant immunosuppressive effect of microglial phagocytosis in fAβ_42-_stimulated N9 cells. Among EP receptors, EP2 was shown to have the highest inhibition in microglial phagocytosis, exhibiting functional activity in response to PGE_2_. Furthermore, incubation of cells with curcumin, a potent immunomodulating therapeutic natural compound in AD, prevents PGE_2_ from suppressing fAβ_42_-elicited phagocytosis, through the inhibition of EP2 activity or its downstream cAMP-PKA-CREB signaling.

It appears that early in the AD process, microglia play a neuroprotective role by promoting Aβ phagocytosis, degradation, and clearance [24]. However, with disease progression, microglia transform to a pro-inflammatory phenotype, lose their ability to clear Aβ, and produce pro-inflammatory cytokines which in turn reduce Aβ uptake and degradation [25]. These distinct effects may be explained by molecular phenotypes and effector functions, depending on the induced signaling pathway of microglial response. Recent studies increasing evidence for the microglial pro-inflammatory responses in the development of AD. It has been reported that the inflammatory and oxidative milieu (LPS or tert-butyl hydroperoxide) significantly downregulates fAβ_42_-induced scavenger receptors in microglial cells [20]. Pro-inflammatory cytokines (IL-1β, TNF-α, and IFN-γ) have been shown to inhibit fAβ-stimulated phagocytosis of fluorescent microspheres in BV-2 microglial cells [3]. However, the exact cytokines act selectively to regulate microglial phagocytosis in AD brain are not fully understood yet. The prominent pro-inflammatory responses in the AD brain are the rapid induction of COX-2 and prostaglandins [5, 26]. In particular, PGE_2_ is of most interest in the development of AD, as it is initially significantly elevated in patients with very early stage or probable AD [27]. Recently, Johansson *et al*. indicated that microglial chemotaxis and Aβ clearance were restored by a knockout microglia-specific PGE_2_ receptor [28]. In the present study, sequential treatment was designed to mimic and investigate the roles of mature Aβ-fibrils and PGE_2_-related inflammation in the AD brain. We validated the fact that treatment with 1 μM fAβ_42_ for 3 and 6 h significantly increased phagocytosis of latex beads in N9 cells, while increased phagocytosis was dramatically reduced by exogenous PGE_2_ or endogenous PGE_2_ induced by LPS. Combined with previous epidemiologic evidence demonstrating that long-term nonsteroidal anti-inflammatory drugs have beneficial effects on reducing AD risk [29, 30], we speculate that pharmacologic agents targeting the anti-inflammatory and pro-phagocytic activities of microglia may be helpful for preventing the inhibitory effect of PGE_2_ on Aβ_42_-induced phagocytosis, which is thus beneficial for suppressing AD development.

Prior work has suggested that PGE_2_, a pivotal immunosignaling agent in models of AD, can exert both detrimental and beneficial effects depending on the function of the EP receptors. EP1 receptor has been reported to aggravate neurotoxicity in AD and cerebral ischemia [31]. EP2 signaling is associated with pro-inflammatory gene upregulation, and the inhibition of beneficial chemokine production and Aβ clearance underlying aging and/or Aβ_42_ accumulation [28]. EP3 might be a pro-inflammatory signal to generate increased Aβ peptides [32]. In contrast, the EP4 receptor has been shown to suppress toxic inflammatory responses to Aβ_42_ and potentiate phagocytosis of Aβ_42_ [33]. Although EP receptors were abundantly expressed in glial cells and could play some role in neuronal interaction, it is important to note the unique function of microglial cells linked to their toxic inflammatory responses and potentiation of phagocytic effects. In this study, the agonists and antagonists of EP1-4 were used to confirm which subtype of the E-prostaniod receptor involved in the regulation of microglial phagocytosis. Our results demonstrated that the reduction effect of PGE_2_ on fAβ_42_-induced phagocytosis appeared to be mainly mediated by the EP2 and EP4 receptor rather than the EP1 and EP3 receptor. Moreover, an increase in cAMP levels were observed in N9 cells incubated with the selective EP2 or EP4 agonists butaprost or L-902,688, respectively, indicating the activation of cAMP by the activity of EP2 and EP4 receptors. These results suggest the participation of EP2 and/or EP4 receptors in fAβ_42_-stimulated N9 cells with exogenously supplied PGE_2_. In an effort to reveal the essential role of microglial EP receptors in the action of PGE_2_, we used EP antagonists and agonists to explore whether EP2 or EP4 mediate the reduction effect of PGE_2_ action in N9 cells. We found that PGE_2_-induced impairement of microglial phagocytosis by fAβ_42_-stimulated N9 cells was prevented by the EP2 antagonist AH6809, but not by the EP4 antagonist GW627368X. Moreover, the EIA assay showed significant abolishment of cAMP levels by the EP2 specific antagonist AH6809, suggesting a potent role of EP2 in mediating PGE_2_ action in N9 cells. Otherwise, a more robust decrease phagocytosis of beads was observed in N9 cells with the pretreatment of EP2 agonist butaprost than the EP4 agonist L-902,688. The immunoblot assay identified a significant increase in EP2 levels in cells treated with both fAβ_42_ and PGE_2_, as compared to those left untreated. However, EP4 expression was unaffected in all groups. Indeed, several lines of evidence point to microglial EP2 activity as a potentially inhibitor of microglial clearance of Aβ and plaques using receptor-knockout animals [7, 9, 34]. Thus, pharmacologic intervention targeting microglial EP2 signaling may provide critical aid to improve Aβ-related microglial phagocytosis in AD brain.

Epidemiologic studies have suggested that diets rich in phenolic compounds may have preventive effects on the development of dementia or AD. Previous animal studies of curcumin, a phenolic yellow curry pigment, has shown its neuroprotective effects in neurodegenerative conditions such as AD [35, 36]. *In vivo* studies also showed that curcumin could prevent Aβ-infusion induced spatial memory deficits, as well as reduced Aβ deposits in Sprague-Dawley rats [37]. Furthermore, transgenic mouse model studies demonstrated that curcumin seems to reduce amyloid deposits in a manner that promotes disaggregation of existing Aβ deposits and prevents aggregation of those newly formed [38–40]. In our previous study, curcumin was proven to ameliorate the defective phagocytic ability of microglia in a pro-inflammatory condition [15]. In support of these, curcumin has been found to prevent the LPS-mediated induction of cyclooxygenase-2 against pro-inflammatory response [41, 42]. Although curcumin is widely accepted as a potent modulator of microglial gene expression involving various adhesion molecules, chemokine, and pro-inflammatory cytokines (including PGE_2_), less gene that associated with the direct regulation of phagocytosis was detected [43]. However, whether a direct association exists between the restoration of microglial phagocytosis and the inhibition of PGE_2_-induced EP receptor subtype activity by curcumin pretreatment remains poorly understood. Here, we examined the phagocytic ability of PGE_2_ and fAβ_42_-stimulated N9 cells with curcumin pre-conditioning. Considered the potential role of PGE_2_-EP2 signaling in AD [7, 9, 34] and the immunomodulator role of curcumin in microglial pro-inflammatory and phagocytic responses [14–15, 36–37, 39], we tested whether curcumin regulate the PGE_2_-mediated depression of microglial phagocytosis through PGE_2_-EP2 signaling in mimicked neuropathologic feature of AD with a hallmark of amount production of fAβ_42_ and PGE_2_ [5–8, 27]. Our results revealed that curcumin alone did not change the phagocytic ability in the fAβ_42_-stimulated N9 cells without the co-treatment of inflammatory mediator PGE_2_. When PGE_2_ plus fAβ_42_-mediated stimulation of microglial phagocytosis, curcumin restored the attenuated phagocytic effect by PGE_2_. These results indicate that curcumin itself cannot improve microglial phagocytosis in our experiment condition, it just work when the microglial phagocytosis impaired with a PGE_2_-related pro-inflammatory response. In support of this, fAβ_42_ is accepted as a weak agonist of inflammatory responses [20], no change of PGE_2_ was observed in the fAβ_42_-stimulated N9 cells. Our results also showed that curcumin effectively reverses the inhibitory effect of PGE_2_ on fAβ_42_-induced phagocytosis, with a parallel increase in EP2 expression. Moreover, our results showed no detectable change of EP4 expression for each condition, including the curcumin-treated group. Thus, these results suggest that PGE_2_-EP2 signaling pathway in microglia might be a pharmacologic target of curcumin for the treatment of AD.

Although the activation of the EP2 receptor by PGE_2_ appears to be correlated to amyloid burden in AD [5–8, 27], the signaling pathways modulated by curcumin that contribute to its efficacy in the promotion of phagocytosis against AD remain unknown. A recent study suggested that upregulation of DOCK2, an upstream regulator of EP2 receptor, contributes to Aβ plaque burden [8]. However, the downstream targets of microglial EP remain elusive. Theoretically, a pivotal role of EP receptor-stimulated, cAMP-dependent effects of PGE_2_ can be explained by the activation of PKA and CREB in a variety of mammalian cells [44]. Moreover, microglial EP2-PKA signaling was found to be critical to establish the pharmacologic function of an active nature component triptolide in the LPS-induced microglial inflammatory responses [45]. In the present study, we delineated the strong suppression of the activation of cAMP, PKA, and CREB by curcumin against the impaired phagocytosis induced by PGE_2_ in fAβ_42_-stimulated N9 cells. Furthermore, our data also showed that pharmacological inhibitor of PKA, H89, restored the phagocytic functions following PGE_2_ action in fAβ_42_-stimulated N9 cells. Our results combined with previous studies, suggest, in partial, the involvement of EP2-PKA effects on the role of curcumin in the restoration of impaired phagocytosis in fAβ_42_-stimulated N9 cells with PGE_2_.

Although our findings indicated that curcumin ameliorated defective microglial phagocytosis via the inhibition of the PGE_2_-EP2-PKA pathway in N9 cells, it is important to note some limitations of the current models we tested. The immortalized murine microglial cell line N9, elicited a profound pro-phagocytic effect in response to acute stimulation with fibrillar Aβ_42_. Notably, *in vivo*, chronic neuroinflammation and the building blocks of a dynamic and complex network of intermediates such as oligomers, fibrils, and plaques are involved in the pathogenic cascade of AD. Herein, just the fibril form of Aβ was occupied to mimic the late stage of AD. This discrepancy is mainly due to the differences between acute *in vitro* responses of curcumin to exogenous PGE_2_ and Aβ_42_ treatment in an immortalized microglial population, and a chronic *in vivo* response in the whole cerebral cortex and hippocampus to long-term complex interaction between endogenous PGE_2_ and Aβ_42_ for several years. Moreover, various inflammatory mediators exist in the AD brain and, besides PGE_2_, most are likely involved in the regulation of the capacity of microglial phagocytosis either directly or indirectly [3]. In this study we only focused on the pharmacological intervention of PGE_2_ by curcumin in N9 cells following acute fibrillar Aβ_42_ stimulation. Hence, despite the pharmacologic outcome of curcumin in our study, the basis for the effects of curcumin over various time course and the involvement of different cytokines in the AD brain remains to be solved. Further experiments are warranted to explore the detailed preventive mechanisms of curcumin underlying neurologic disorders.

In summary, we demonstrated that curcumin restored the inhibitory effect of PGE_2_ on phagocytosis of Aβ_42_-stimulated N9 microglial cells. Moreover, curcumin ameliorated phagocytic abilities via inhibition of the PGE_2_-EP2 system with partial involvement in a cAMP-PKA-dependent manner. Further studies targeting the precise downstream signaling of the EP2 receptor hold promise to reveal the therapeutic mechanisms of curcumin for AD and other neurodegenerative diseases.

## Supporting Information

S1 Fig Effect of DMSO on phagocytosis in N9 cells. N9 cells were stimulated with DMSO (0.1 and 0.2%) for 3 and 6 h. Then, cells were subjected to a 1 h process of phagocytosis of fluorescent-labeled latex beads (0.00125%) for the phagocytosis assay on a flow cytometer. The results are expressed as % of the untreated control, and are presented as means ± SEM of three independent experiments. Statistical significance was determined by one-way ANOVA followed by Tukey’s test. con, control; DMSO, dimethylsulfoxide.
(TIF)

S2 Fig Dose response curves of antagonists of PG receptors EP1-4 in N9 cells. N9 cells were pretreated with dosage of antagonists of PG receptors EP1-4. Then, cells were stimulated with fAβ_42_ (1 μM) in the presence or absence of exogenous PGE_2_ (5 μM) for 3 h. Subsequently, cells were subjected to a 1 h process of phagocytosis of fluorescent-labeled latex beads (0.00125%). Average fluorescence intensity of latex beads ingested and normalized phagocytosis analysis were estimated for each group using FACS analysis. The results are expressed as % of the untreated control, and are presented as means ± SEM of three independent experiments. Statistical significance was determined by one-way ANOVA followed by Tukey’s test.*P < 0.05 vs the untreated control group; ^#^*P* < 0.05 vs the fAβ_42_-stimulated group. con, control; PGE_2_, prostaglandin E2; fAβ_42_, fibrillar Aβ peptide (1–42); GW8, GW848687X; AH, AH6809; L-7, L-798106; GW6, GW627368X.
(TIF)

S3 Fig Dose response curves of agonists of PG receptors EP1-4 in N9 cells. N9 cells were pretreated with dosage of agonists of PG receptors EP1-4. Then, cells were stimulated with fAβ_42_ (1 μM) in the presence or absence of exogenous PGE_2_ (5 μM) for 3 h. Subsequently, cells were subjected to a 1 h process of phagocytosis of fluorescent-labeled latex beads (0.00125%). Average fluorescence intensity of latex beads ingested and normalized phagocytosis analysis were estimated for each group using FACS analysis. The results are expressed as % of the untreated control, and are presented as means ± SEM of three independent experiments. Statistical significance was determined by one-way ANOVA followed by Tukey’s test.*P < 0.05 vs the untreated control group; ^#^*P* < 0.05 vs the fAβ_42_-stimulated group. con, control; PGE_2_, prostaglandin E2; fAβ_42_, fibrillar Aβ peptide (1–42); PTPE2, 17-phenyl trinor Prostaglandin E2; bu, butaprost; su, sulprostone; L-9, L-902,688.
(TIF)

S4 Fig Effect of fAβ_42_ and curcumin on the production of PGE_2_ in N9 cells. N9 cells were pretreated with or without curcumin (10 μM) for 30 min prior to fAβ_42_ (1 μM) treatment for 3 h. Enzyme immunoassay of PGE_2_ was performed as described in Methods. Experiments were performed with three replicates for each experimental condition. Data are presented relative to control and are presented as means ± SEM of five independent experiments. Statistical significance was determined by two-way ANOVA followed by Tukey’s test. con, control; PGE_2_, prostaglandin E2; fAβ_42_, fibrillar Aβ peptide (1–42); Cur, curcumin.
(TIF)

S5 Fig Dose response curves of inhibitor and activator of PKA in N9 cells. N9 cells were pretreated with dosage of PKA inhibitor H89 or PKA activator 6-Bnz-cAMP for 30 min. Then, cells were stimulated with fAβ_42_ (1 μM) in the presence or absence of exogenous PGE_2_ (5 μM) for 3 h. Subsequently, cells were subjected to a 1 h process of phagocytosis of fluorescent-labeled latex beads (0.00125%). The results are expressed as % of the untreated control, and are presented as means ± SEM of three independent experiments. Statistical significance was determined by one-way ANOVA followed by Tukey’s test.*P < 0.05 vs the untreated control group; ^#^*P* < 0.05 vs the fAβ_42_-stimulated group. con, control; PGE_2_, prostaglandin E2; fAβ_42_, fibrillar Aβ peptide (1–42); 6-Bnz-cAMP, Adenosine 3ʹ,5ʹ-cyclic Monophosphate, N^6^-Benzoyl-, Sodium Salt.
(TIF)
