# Supplementary figures and images for: Curcumin Ameliorates the Reduction Effect of PGE2 on Fibrillar β-Amyloid Peptide (1-42)-Induced Microglial Phagocytosis through the Inhibition of EP2-PKA Signaling in N9 Microglial Cells
Source: PLoS One. 2016 Jan 29;11(1):e0147721. doi: 10.1371/journal.pone.0147721 (PMC4732694; doi:10.1371/journal.pone.0147721)

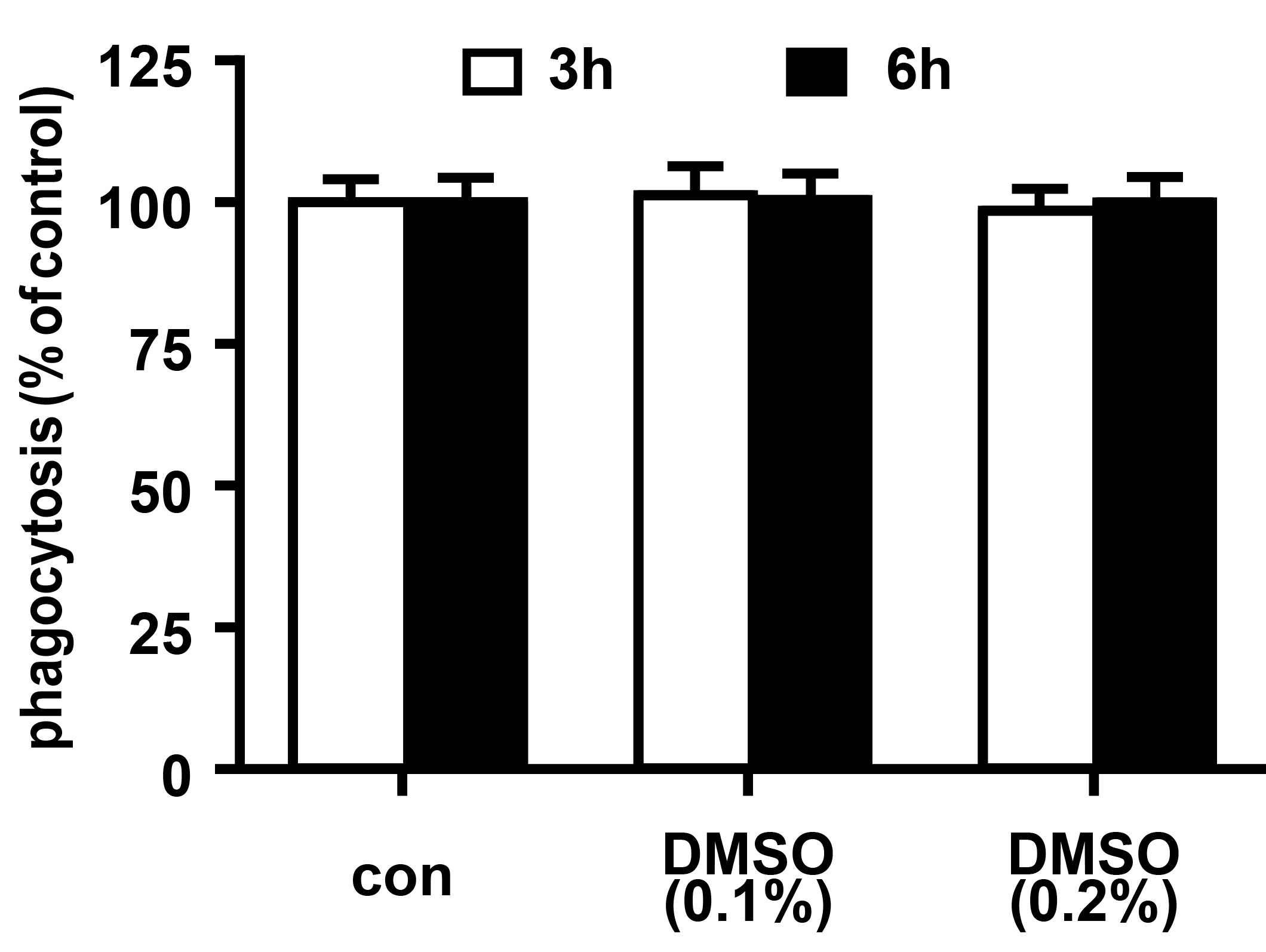

Supplement: S1 Fig — N9 cells were stimulated with DMSO (0.1 and 0.2%) for 3 and 6 h. Then, cells were subjected to a 1 h process of phagocytosis of fluorescent-labeled latex beads (0.00125%) for the phagocytosis assay on a flow cytometer. The results are expressed as % of the untreated control, and are presented as means ± SEM of three independent experiments. Statistical significance was determined by one-way ANOVA followed by Tukey’s test. con, control; DMSO, dimethylsulfoxide. (TIF) [file pone.0147721.s001.tif]

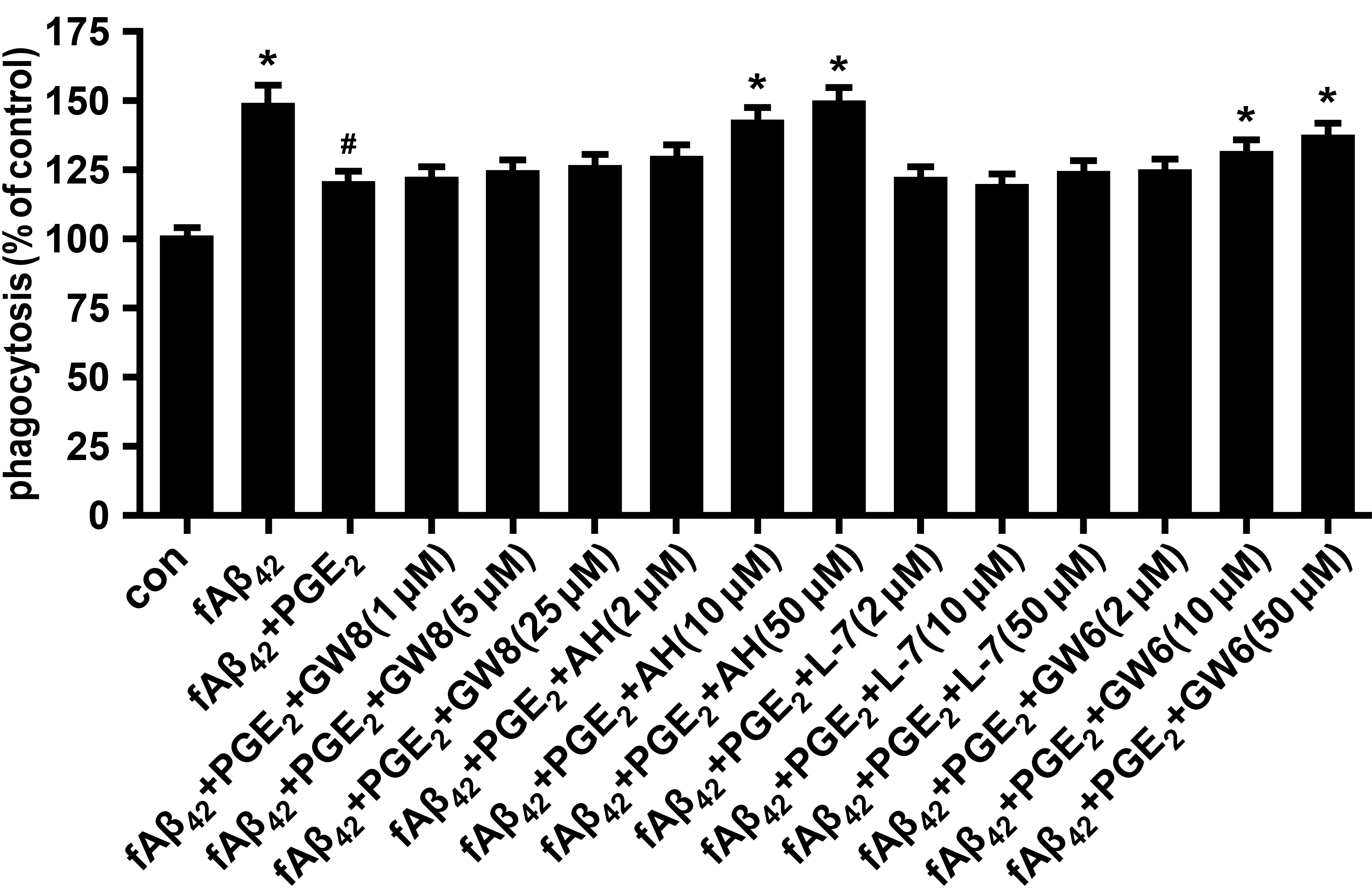

Supplement: S2 Fig — N9 cells were pretreated with dosage of antagonists of PG receptors EP1-4. Then, cells were stimulated with fAβ42 (1 μM) in the presence or absence of exogenous PGE2 (5 μM) for 3 h. Subsequently, cells were subjected to a 1 h process of phagocytosis of fluorescent-labeled latex beads (0.00125%). Average fluorescence intensity of latex beads ingested and normalized phagocytosis analysis were estimated for each group using FACS analysis. The results are expressed as % of the untreated control, and are presented as means ± SEM of three independent experiments. Statistical significance was determined by one-way ANOVA followed by Tukey’s test.*P < 0.05 vs the untreated control group; #P < 0.05 vs the fAβ42-stimulated group. con, control; PGE2, prostaglandin E2; fAβ42, fibrillar Aβ peptide (1–42); GW8, GW848687X; AH, AH6809; L-7, L-798106; GW6, GW627368X. (TIF) [file pone.0147721.s002.tif]

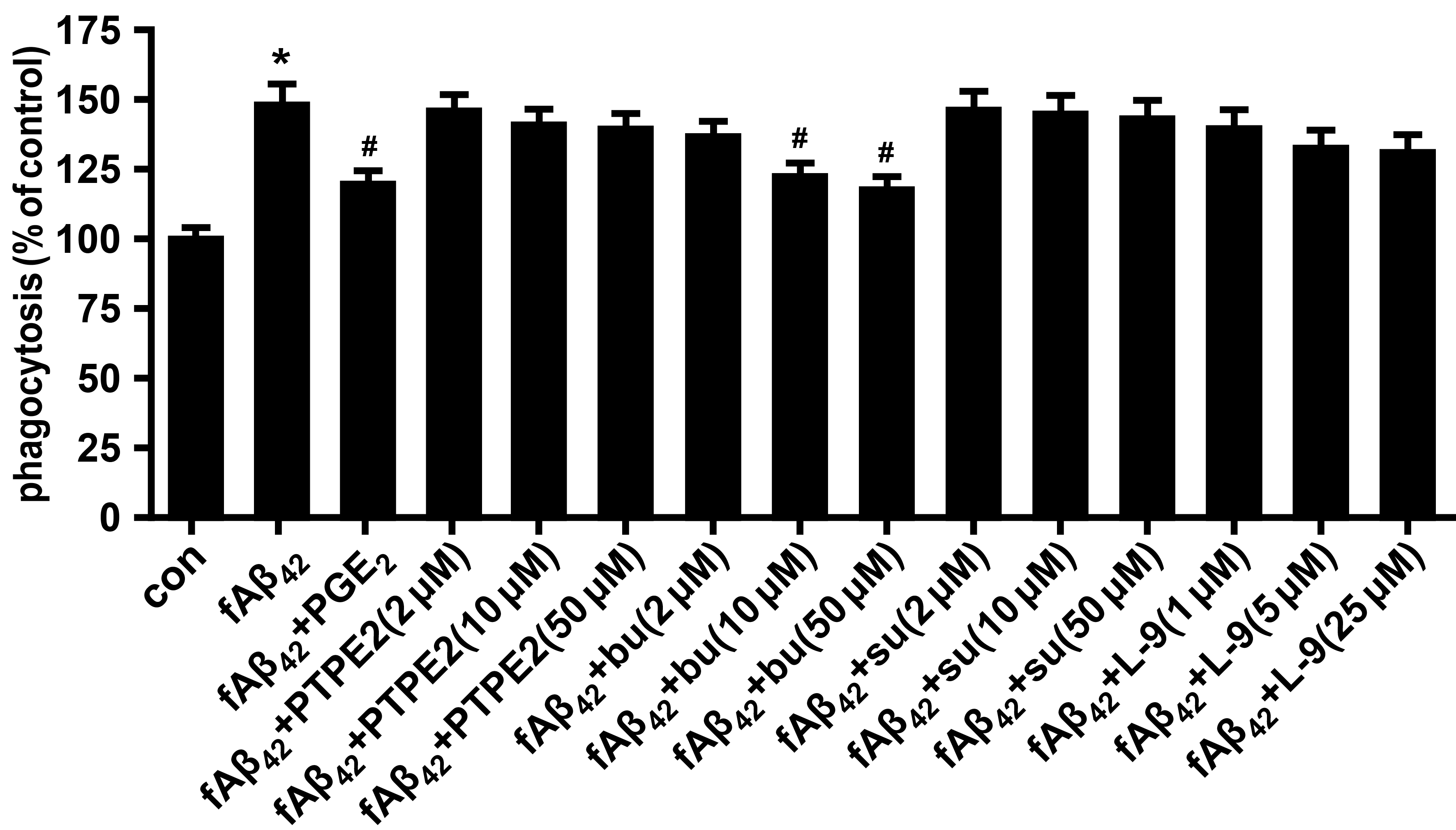

Supplement: S3 Fig — N9 cells were pretreated with dosage of agonists of PG receptors EP1-4. Then, cells were stimulated with fAβ42 (1 μM) in the presence or absence of exogenous PGE2 (5 μM) for 3 h. Subsequently, cells were subjected to a 1 h process of phagocytosis of fluorescent-labeled latex beads (0.00125%). Average fluorescence intensity of latex beads ingested and normalized phagocytosis analysis were estimated for each group using FACS analysis. The results are expressed as % of the untreated control, and are presented as means ± SEM of three independent experiments. Statistical significance was determined by one-way ANOVA followed by Tukey’s test.*P < 0.05 vs the untreated control group; #P < 0.05 vs the fAβ42-stimulated group. con, control; PGE2, prostaglandin E2; fAβ42, fibrillar Aβ peptide (1–42); PTPE2, 17-phenyl trinor Prostaglandin E2; bu, butaprost; su, sulprostone; L-9, L-902,688. (TIF) [file pone.0147721.s003.tif]

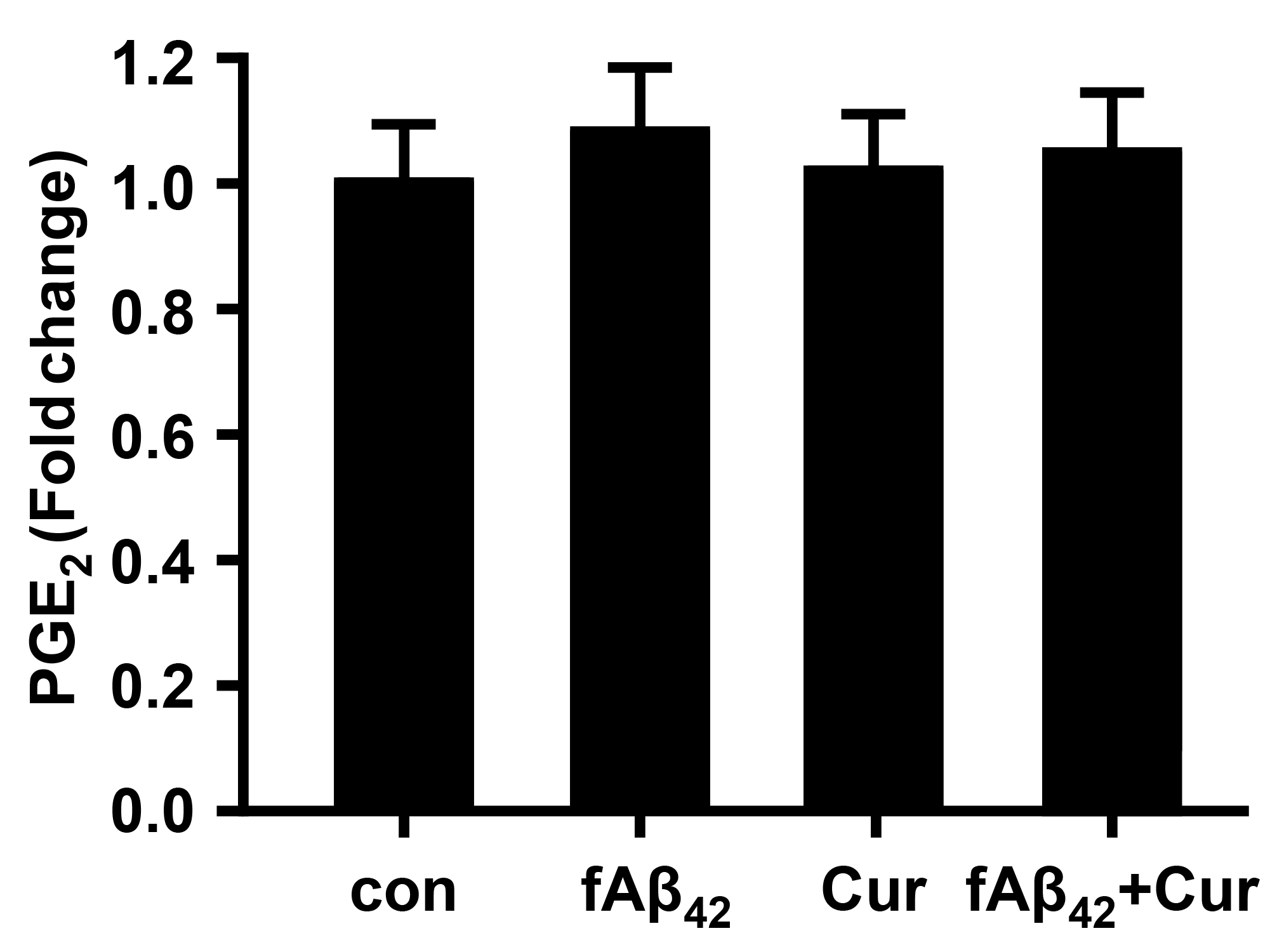

Supplement: S4 Fig — N9 cells were pretreated with or without curcumin (10 μM) for 30 min prior to fAβ42 (1 μM) treatment for 3 h. Enzyme immunoassay of PGE2 was performed as described in Methods. Experiments were performed with three replicates for each experimental condition. Data are presented relative to control and are presented as means ± SEM of five independent experiments. Statistical significance was determined by two-way ANOVA followed by Tukey’s test. con, control; PGE2, prostaglandin E2; fAβ42, fibrillar Aβ peptide (1–42); Cur, curcumin. (TIF) [file pone.0147721.s004.tif]

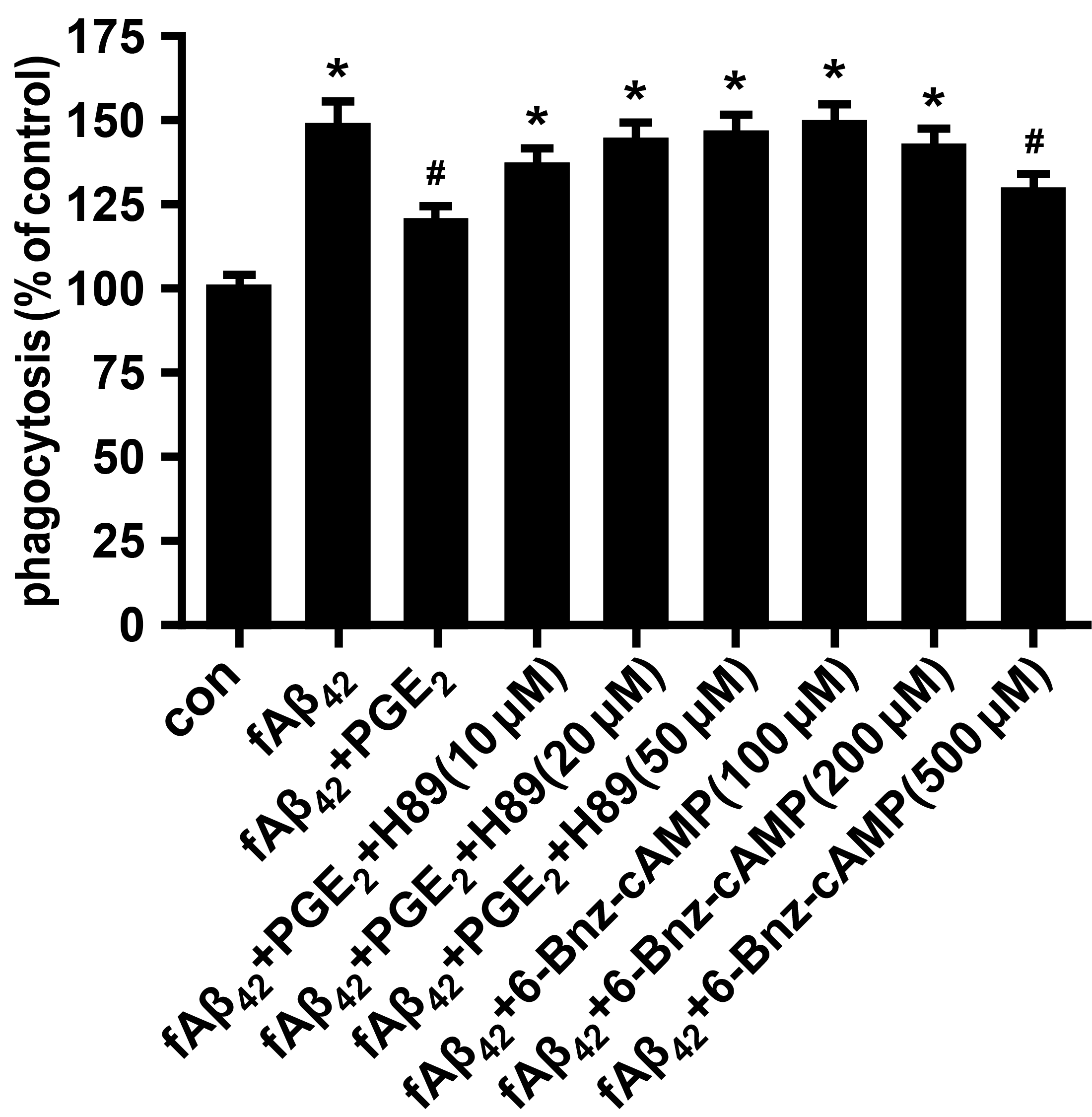

Supplement: S5 Fig — N9 cells were pretreated with dosage of PKA inhibitor H89 or PKA activator 6-Bnz-cAMP for 30 min. Then, cells were stimulated with fAβ42 (1 μM) in the presence or absence of exogenous PGE2 (5 μM) for 3 h. Subsequently, cells were subjected to a 1 h process of phagocytosis of fluorescent-labeled latex beads (0.00125%). The results are expressed as % of the untreated control, and are presented as means ± SEM of three independent experiments. Statistical significance was determined by one-way ANOVA followed by Tukey’s test.*P < 0.05 vs the untreated control group; #P < 0.05 vs the fAβ42-stimulated group. con, control; PGE2, prostaglandin E2; fAβ42, fibrillar Aβ peptide (1–42); 6-Bnz-cAMP, Adenosine 3ʹ,5ʹ-cyclic Monophosphate, N6-Benzoyl-, Sodium Salt. (TIF) [file pone.0147721.s005.tif]
